# Supplementary material for: Chromothripsis during telomere crisis is independent of NHEJ, and consistent with a replicative origin
Source: Genome Res. 2019 May;29(5):737–49. doi: 10.1101/gr.240705.118 (PMC6499312; doi:10.1101/gr.240705.118)
Supplement: Supplemental Material [file supp_gr.240705.118_Supplemental_file_1.zip › contigs/annotated_contigs/DB93/contig.2.DB93_length_588_mean_cov_9.0119047619.docx]

**DB93_length_588_mean_cov_9.0119047619**

```
TGTCACAAAAGCCCATGGACAGAGCACAGGAAAGACAGGCACATAACCTGAATATCAGCTTCAGTGGTATGACCCAATGCCTCCTGTGA
 >chr7_gl000195_random:15162-15621 - E=1e-243
GCGTTCCAAGGCAGGAGAGGAGACTCACATTACCTGTGTGCAAGACCCAGTGATACGTCACACGGAGGAGTACCACTGTCTTGCATATT

GTGTAAACTATGGTAGAGAAATTGTCACCACAGGGCTCGCCACACTGGTGAGATTATACTTCTCAGATTCACACCACACCAATATTCAG

GATGGTCTCTATCACATGTGGAGAGAGCCCACTCTTGAGGTCCTGAATTACACATGCAGACATAGTCCACAACTGGGATTCTGACTTTC

ATATGTGAACATCCAGCCACAGGTGGGATGGTGACTCATTTTTAAACGCAGCTCATAGGCAGTTAAGAACTCTTATTTGGACCCATCCA

AGTAGAAAG|ATGTT|CGCAGTTGGGGGCTCAAATCACTGACAGGAAGAAGCTGTACTCTGGGCAATGATGACACCCCTTCCTTAAATT
          >chr17:47899202-47899338 + E=4e-64
AAAAAAAAAAAAGGCAAACCCATGTATCAAAGATTGTTTAATGAAAATGCAGTTTT
```
